# Supplementary material for: The causal relationship between the human gut microbiota and pyogenic arthritis: a Mendelian randomization study
Source: Front Cell Infect Microbiol. 2024 Nov 26;14:1452480. doi: 10.3389/fcimb.2024.1452480 (PMC11629706; doi:10.3389/fcimb.2024.1452480)
Supplement: Supplementary file 1 [file DataSheet1.zip › mendelian test/ebi-a-GCST90027684.csv_forest.pdf]

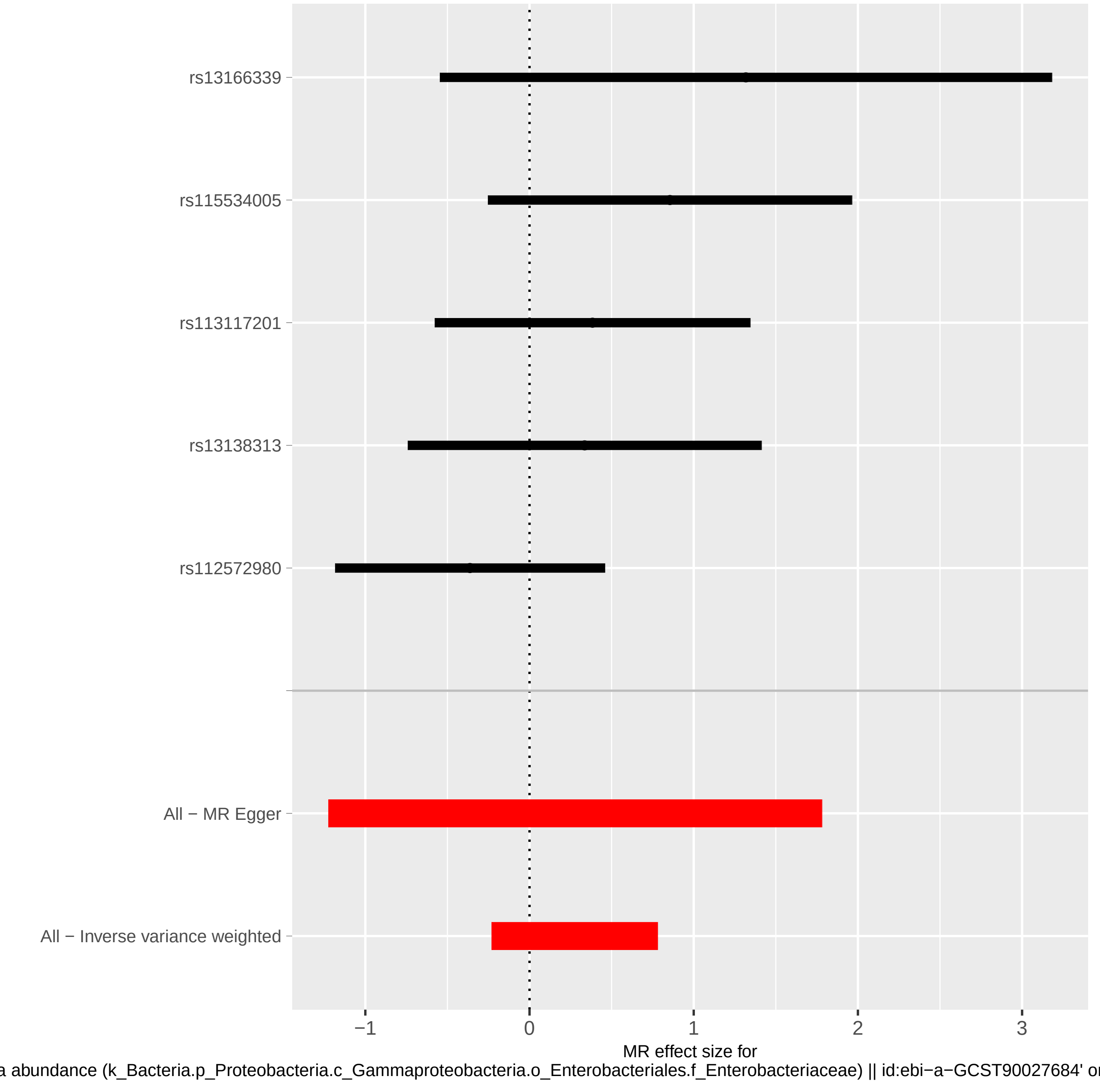

rs13166339
rs115534005
rs113117201
rs13138313
rs112572980
All − MR Egger
All − Inverse variance weighted
−1
0
1
2
3
MR effect size for
a abundance (k_Bacteria.p_Proteobacteria.c_Gammaproteobacteria.o_Enterobacteriales.f_Enterobacteriaceae) || id:ebi−a−GCST90027684' or
